# Prevention of uropathogenic *E. coli* biofilm formation by hydrophobic nanoparticle coatings on polymeric substrates†

**DOI:** 10.1039/d3lf00241a

**Published:** 2024-03-20

**Authors:** Stefanie Dietl, Padryk Merkl, Georgios A. Sotiriou

**Affiliations:** a Department of Microbiology, Tumor and Cell Biology, Karolinska Institutet SE-17177 Stockholm Sweden georgios.sotiriou@ki.se

## Abstract

Biofilms in infections are a major health-care challenge and strategies to reduce their formation on medical devices are crucial. Fabrication of superhydrophobic coatings based on hydrocarbon adsorption on rare-earth oxides constitutes an attractive strategy, but their capacity to prevent biofilm formation has not been studied. Here, we explore a scalable and reproducible nanofabrication process for the manufacture of such superhydrophobic coatings and study their antibiofilm activity against clinically-relevant uropathogenic *E. coli*. These coatings reduce bacterial biofilm formation and prevent biofouling with potential applications preventing medical device related infections.

## Abstract 2

Flame-made rare-earth nanostructured coatings are deposited *in situ* on catheter-mimicking surfaces and subsequently rendered superhydrophobic in a controlled way by hydrocarbon adsorption, preventing the growth of uropathogenic *E. coli* biofilms.

## Graphical abstract

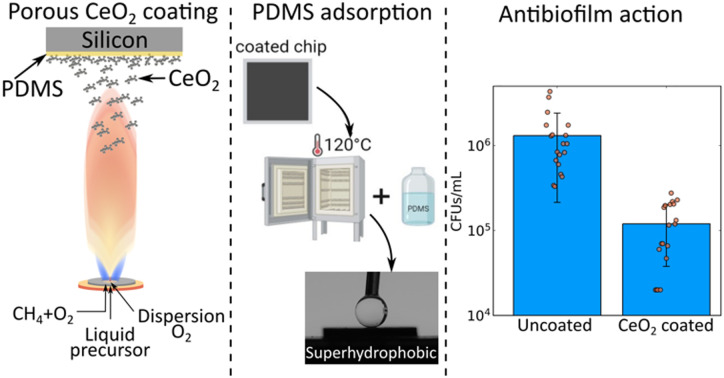

Bacterial biofilm formation on human tissue and medical devices, such as catheters and sutures, constitutes up to 80% of microbial infections.^1,2^ The ability of biofilms to resist both clearing by the immune system and antimicrobial treatments calls for new antibiofilm strategies. The first stage of biofilm formation occurs when bacteria growing in a planktonic state adhere to a surface and form polymicrobial aggregates. Subsequently, as these microcolonies grow and mature, they produce extracellular polymeric substances (EPSs), which are the main components of biofilms.^1,3^ These EPSs are responsible for the adhesion and cohesion of the biofilm, allowing the formation of a three-dimensional architecture.^3^ These polymicrobial communities also contribute to the survival of the microorganisms through altered metabolic activities and genetic adaptations.^4^ EPSs consist mainly of water, contributing to approximately 97% of the total volume.

Abiotic surfaces of medical devices are particularly vulnerable to biofilm formation. Typically, if a biofilm forms on a medical device, the device either has to be removed or, if this is not possible, the patient has to undergo long-term aggressive antibiotic treatment.^5^ Therefore, prevention of biofilm formation on medical device surfaces is an attractive prospect. Various strategies have been proposed to combat biofilm formation, however, these typically target mature biofilms.

Preventing the attachment of bacteria and the subsequent biofilm formation can improve clearance of the bacteria by the host immune system. Moreover, this would avoid the necessity of overcoming treatment challenges associated with mature biofilms. This approach can therefore avoid the excessive use of antibiotics and help reduce the risks of antibiotic resistance development. One such strategy to prevent the attachment is the application of antifouling or antibiofilm surfaces.^5,6^

The main factors that promote biofilm formation are the biological environment and the physical properties of the surface. Two of the most important surface properties determining biofilm formation are the hydrophilicity and the roughness of the surface. Depending on the scale and surface chemistry of the surface, roughness can promote or reduce biofilm formation.^7^ An appropriately designed surface can trap air bubbles in a rough surface structure and thereby reduce the liquid-exposed surface area for bacterial attachment. Alternatively, by reducing the surface energy, the adhesion forces to the surface can be reduced, preventing bacterial attachment. These are the critical characteristics of many hydrophobic nanocoatings that prevent the attachment of microorganisms.^8^ Hydrophobic surfaces against biofilm formation have received much attention in recent years due to their low cost, biocompatibility and facile preparation, rendering them a promising strategy to battle biofilm formation on medical devices.

A powerful way to produce inorganic nanoparticle coatings is through flame spray pyrolysis (FSP).^9^ This technology allows for facile production of single- or multi-component nanoparticles with controlled size and composition.^10^ Moreover, direct nanoparticle deposition can be performed in a single-step to achieve highly porous nanocoatings.^11,12^ Nanoparticle coatings by FSP have been extensively used for biomedical applications, with demonstrated efficacy as antibacterial surfaces and for bioanalyte detection.^13,14^ This includes the use of hydrophilic cerium dioxide coatings capable of detecting bacterial hydrogen peroxide production.^15^ Flame-made hydrophobic and superhydrophobic coatings have also been demonstrated, such as titanium dioxide as a superhydrophobic paper-board coating or the single-step synthesis and hydrophobic functionalisation of Mn_3_O_4_, ZnO, and TiO_2_.^16–18^ However, the intrinsic catalytic properties of these particles can raise some concerns for their long term use in biomedicine due to the production of reactive oxygen species.

One attractive class of materials for inorganic hydrophobic coatings are rare earth oxides. Many of these oxides such as CeO_2_ were thought to be intrinsically hydrophobic.^19^ However, subsequent studies suggested that these observations were largely due to atmospheric hydrocarbon adsorption.^20^ This ability of REOs to adsorb hydrophobic organic molecules from the vapour phase can allow the controlled production of highly porous hydrophobic REO coatings. Organic/inorganic hybrid systems consisting of polydimethylsiloxane (PDMS) and CeO_2_ have been shown to exhibit superhydrophobicity. This coating was sprayed onto the desired substrate and subsequently cured and demonstrated a contact angle of ∼158°.^21^ This coating had good stability in a simulated outdoor environment, maintaining long-term superhydrophobicity. However, the coating was not evaluated for anti-fouling properties or its potential for biofilm inhibition.

The combination of CeO_2_ nanoparticles and PDMS for medical device superhydrophobic surfaces is the key topic of the work presented here. CeO_2_ nanoparticle coatings were deposited directly by FSP onto catheter-mimicking surfaces (30 μm thick PDMS-coated Si substrates 5 × 5 mm). Subsequent vapour phase deposition of silicone oil was performed to render the coatings superhydrophobic as described by Mamedov *et al.*^22^ To control the vapour phase deposition of hydrophobic molecules onto the CeO_2_ surface, the substrates are placed inside a sealed vessel together with a vial of silicone oil in a furnace at 120 °C for <2 h. The elevated temperatures enable an increased concentration of silicone oil in the vapour phase which can subsequently deposit onto the CeO_2_ surface due to the high affinity of CeO_2_ towards organic molecules (please see the ESI,† Fig. S1 for an overview of this process).

The flame-made CeO_2_ nanoparticles had the typical polygonal geometry under TEM, as shown in Fig. 1a.^23^ Powder diffraction patterns were collected confirming the formation of cubic CeO_2_ with the space group *Fm*3̄*m* (Fig. 1b). The crystallite size determined by Rietveld refinement across the whole diffractogram was 10 nm, in agreement with the literature for particles made at similar FSP process conditions.^15,23^

**Fig. 1:**
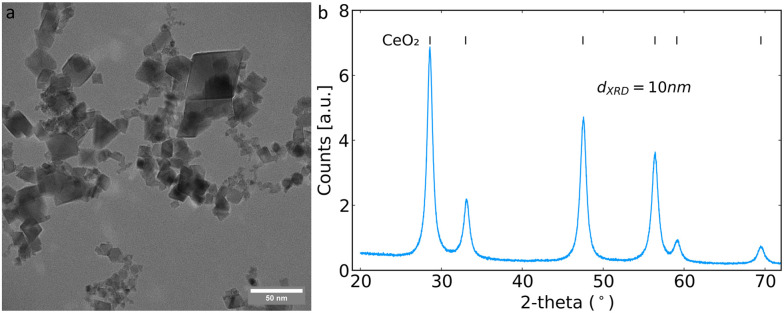
a) TEM image and b) XRD diffractogram of the synthesised CeO_2_ nanoparticles. Vertical lines in b) denote the peaks assigned to the known CeO_2_ structure, and the crystallite size calculated from Rietveld refinement performed on the whole diffractogram is included as an annotation.

The as-deposited films for 30 s on the PDMS-coated substrates achieved a spatially homogeneous and uniform CeO_2_ coating with thickness of ∼20 μm as shown in the top-view (a) and side-view SEM images (d) in Fig. 2. The as-deposited coatings were hydrophilic and unstable to immersion in water. This is demonstrated by the instability of the CeO_2_ coating, as shown in Fig. 2b and e, after immersion in ultrapure water. In contrast, the silicone oil vapour deposition on the as-deposited nanoparticle films renders the substrates stable to immersion in water, as shown in Fig. 2c and f, with little or no film detachment after immersion in water and reduced film restructuring. Thus, this process renders the nanoparticle coatings mechanically stable and allows for their incubation in aqueous solutions, such as bacterial cultures, as investigated later on.

**Fig. 2:**
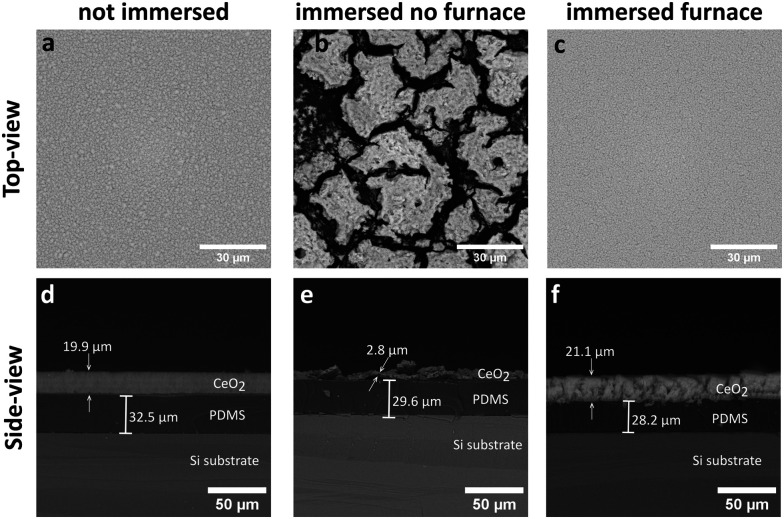
Side-view and top-view SEM images of the as-synthesised CeO_2_ coatings (a and d) and after immersion in water of untreated (b and e) and silicone oil-treated (c and f) CeO_2_ coatings.

The contact angle measurement of the as-deposited coatings is less than 20°, as shown in Fig. 3a. Due to the high temperatures and oxidising nature of the flame used to prepare the CeO_2_ nanoparticles, the surface of the CeO_2_ immediately after synthesis can be expected to be clean and free from contaminating hydrophobic molecules. This result is therefore in good agreement with the results of a previous work demonstrating the hydrophilic nature of bare CeO_2_ nanoparticles.^20^

**Fig. 3:**
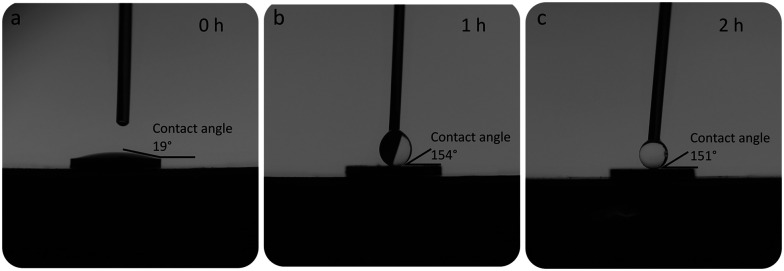
Contact angle measurements of (a) the as-synthesised coatings (0 h) and after silicone oil adsorption for 1 h (b) and 2 h (c).

However, the silicone oil vapour deposition renders the coatings hydrophobic as can be seen from the contact angle measurements shown in Fig. 3b and c. After 1 h in the furnace, a superhydrophobic coating is achieved with little subsequent change in contact angle for longer incubation periods. Moreover, these silicone oil functionalized coatings retain their superhydrophobicity after immersion in water. This immersion stability and retention of the superhydrophobicity was verified up to 1 week of immersion in ultrapure water and Luria broth bacterial growth medium. To validate the affinity of this vapour silicone oil deposition process towards rare earth oxides, we compared the effect on flame-deposited amorphous SiO_2_ nanoparticle films onto PDMS substrates that showed no hydrophobic properties for up to 4 h of vapour phase silicone oil deposition (ESI,† Fig. S2). In contrast, the CeO_2_ coatings achieve superhydrophobicity after only 1 h and the contact angles observed were consistently above 150° with values in good agreement with the results of Oh *et al.*, who achieved contact angle values between 150° and 160° with PDMS/CeO_2_, and of Ashok *et al.*, who achieved a contact angle of 157° with fluoro functionalised silica nanoparticle coatings.^18,21^

After successfully achieving superhydrophobic coatings, their antibiofilm capabilities were tested against a clinically-relevant strain of uropathogenic *E. coli* isolated from a patient with a urinary tract infection that can form biofilms on silicone urinary catheters. The hydrophobic surfaces were incubated in Luria broth growth medium with *E. coli* for 24 hours at 37 °C from a starting optical density at 600 nm of 0.05. The substrates were then removed from the medium and washed with PBS to remove unattached or loosely attached bacteria.^24^ The remaining attached bacteria were classified as a biofilm and plated onto Luria agar for quantification of colony forming units (CFU mL^−1^). This therefore gives a measure of the amount of biofilm formed on the substrates. The bare Si substrate is an extremely smooth surface and shows slightly lower biofilm formation compared with the PDMS coated substrate. The superhydrophobic CeO_2_ coating showed a clear inhibition of biofilm formation, with a greater than one log reduction of bacterial growth compared with the PDMS coated substrate control (Fig. 4a). This corresponds to a reduction of more than 90% of the bacterial biofilm load. This can be put in the context of a recent study by Fu *et al.*, which achieved a reduction of bacterial growth between 22 and 99%, however, their superhydrophobic coating was in combination with an antibacterial surface.^25^ Our study achieved a growth reduction of more than 90% without having an intrinsic antibacterial effect. The application of fluoro functionalised silica hydrophobic coatings was able to achieve a 99.85% reduction in bacterial attachment.^18^

**Fig. 4:**
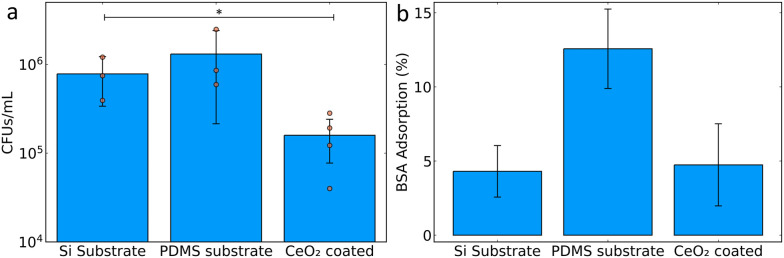
(a) Measurements of biofilm formation and (b) bovine serum albumin adsorption on substrates with and without CeO_2_ coating after silicone oil adsorption. Data in (a) from at least three biological triplicates (mean values shown as orange circles), with each biological replicate consisting of at least four technical replicates, total *n* ≥ 12. *p < 0.05 (equal variance *t*-test).

Additionally, an important consideration for both the longevity of the anti-biofilm activity and immunogenic potential of such superhydrophobic surfaces is the biofouling caused by protein adsorption.^26^ To assess this, bare Si, PDMS-coated and superhydrophobic CeO_2_-coated PDMS substrates were incubated together with bovine serum albumin (BSA) and the adsorbed BSA was quantified by the bicinchoninic acid assay. Bare Si substrates showed very little adsorption of BSA, whereas catheter-mimicking PDMS coated substrates demonstrated BSA adsorption above 10% of the available amount. However, the superhydrophobic CeO_2_-coated substrates had a clear reduction in BSA adsorption to below 5%. This demonstrates the low level of protein adsorption achieved, suggesting a reduced risk for protein fouling effects leading to unwanted immunological and blood clotting reactions.

These results demonstrated for the first time the potential of flame-deposited CeO_2_ coatings with adsorbed silicone oils in a biomedical context. Flame-made inorganic nanoparticle coatings exhibit extremely high porosity (>95%) and by leveraging the vapour phase adsorption of silicone oils on CeO_2_, this high porosity could be exploited in a facile manner without affecting the film structure. The combination of biocompatible materials CeO_2_ and PDMS to produce immersion stable superhydrophobic coatings was shown to reduce the formation of biofilms of a clinically-relevant uropathogenic *E. coli* strain. The nanostructured coatings retain their stability when immersed in aqueous solutions and bacterial cell culture media, enabling the antibiofilm experiments. However, further experiments regarding the durability of the coatings are warranted both *in vitro* and *in vivo*. This approach taken here of preventing the initial attachment of bacteria to a surface does not rely on antibacterial agents and thus has much reduced propensity for the formation of antibacterial resistance. The coatings produced were immersion stable over long periods (>1 week) in even the protein rich Luria growth medium and retained the superhydrophobic properties over this period. An assessment of bovine serum albumin adsorption onto the immersed substrates revealed less than 5% adsorption on the superhydrophobic coatings, suggesting a favourable physiological reaction without severe immunological side effects, prompting for additional *in vitro* and *in vivo* immunological studies, as well as evaluation of multiple bacterial strains. This research provides the framework for the development of antibiofilm nanoparticle coatings on polymer-based devices that mimic catheter surfaces.

## Conflicts of interest

There are no conflicts to declare.

## Supplementary Material

LF-001-D3LF00241A-s001
