# Supplementary material for: Prevention of uropathogenic E. coli biofilm formation by hydrophobic nanoparticle coatings on polymeric substrates
Source: RSC Appl Interfaces. 2024 Mar 20;1(4):667–70. doi: 10.1039/d3lf00241a (PMC11231686; doi:10.1039/d3lf00241a)
Supplement: LF-001-D3LF00241A-s001 [file LF-001-D3LF00241A-s001.pdf]

Electronic Supplementary Material (ESI) for RSC Applied Interfaces.
This journal is © The Royal Society of Chemistry 2024

Supporting information (SI) for:

Prevention of uropathogenic *E. coli* biofilm formation by hydrophobic nanoparticle coatings on polymeric substrates

Stefanie Dietl†, Padryk Merkl† and Georgios A. Sotiriou*

Department of Microbiology, Tumor and Cell Biology, Karolinska Institutet, SE-17177 Stockholm, Sweden.

† These authors contributed equally.

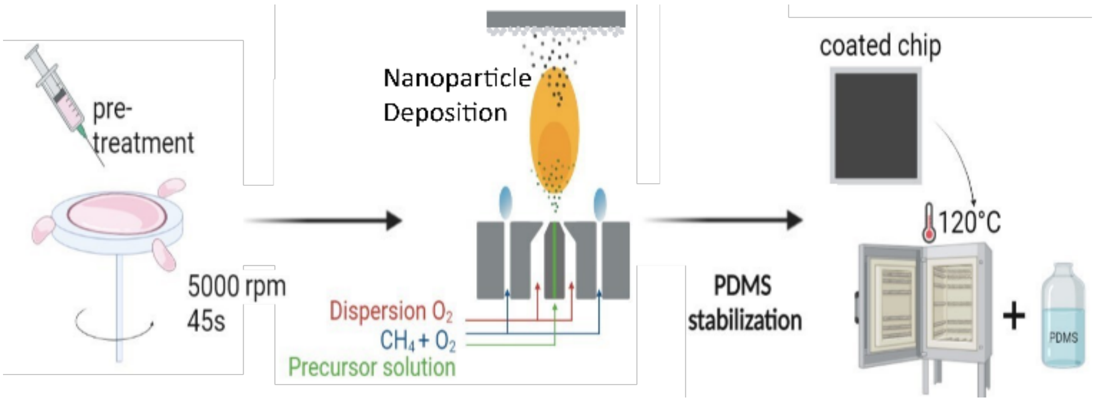

Figure S1: Overview of the procedure for preparation of the superhydrophibic CeO_2 substrates used here. First a PDMS layer is spin-coated onto a silicon substrate. This PDMS coated substrate is then placed on a water-cooled holder above the flame spray pyrolysis burner and a coating of CeO_2 nanoparticles is formed. Silicone oil is deposited onto the CeO_2 coating by vapour phase deposition inside a furnace heated to 120 °C.

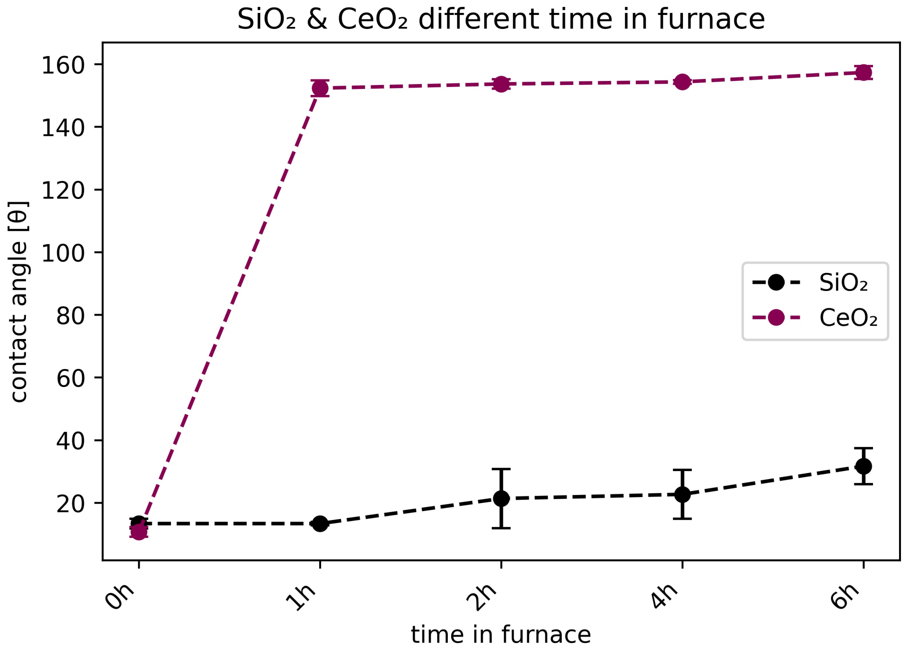

Figure S2: Contact angles measured on substrates coated with SiO_2 (black) or CeO_2 (purple) exposed to silicone oil vapour for different durations.

Materials & Methods

Pre-treatment of silicon substrates

To generate PDMS coated silicon substrates a thin layer of PDMS (Sylgard 184, Dow Chemicals) was spin-coated before deposition with FSP. A 10:1 mixture of sylgard 184 (Dow Chemicals) and curing agent (Dow Chemicals) was added to the Si substrate before spin coating with an acceleration of 500 rpm/s at a speed of 5000 rpm for 45 s with a WS-650MZ-23NPPB spin coater (Laurell).

Deposition and Characterization of nanoparticles

CeO_2 nanoparticles were deposited on PDMS coated Si substrates by FSP. Cerium (III) ethylhexanoate (49 % in 2-ethylhexanoic acid, Thermo Fisher) was dissolved in equal parts Xylene (99 %, Sigma) and 2-ethylhexanoic (99 %, Sigma) acid to achieve a 0.1 M solution. The respective solution was then fed through a syringe pump (New Era Pump Systems, Inc.) at a rate of 10 mL/min and dispersed in 5 L/min of oxygen (99.5 %, AGA Gas AB). The ignition of the flame was achieved by pilot flame consisting of methane and oxygen with a flow rate of 1.5 and 3.2 L/min, respectively (both gases 99.5 %, AGA Gas AB). The Si substrates were attached to a water-cooled holder (16 °C) 16 cm above the nozzle for a deposition of 30 s. Si substrates pre-treated with PDMS were cured at 150 °C for 15 min after deposition. The thickness of the coatings was analysed via scanning electron microscope (Phenom Pharos).

Transformation to superhydrophobicity

After the CeO_2 deposition, the substrates were exposed to a silicone oil rich environment in order to transform them into a superhydrophobic coating via hydrocarbon adsorption. To achieve this, the coated substrates were added into a beaker together with a vial containing silicone oil and the beaker was covered with aluminium foil. The beaker was then transferred to the furnace (Carbolite, Gero) at 120 °C for 2 hours. The contact angles were measured with a custom 3D-printed Contact Angle Goniometer.

Biofilm growth and characterisation

A strain of *E. coli* (HVM52) that was isolated from a catheter associated urinary tract infection was cultured in lysogeny broth (LB) medium at 37 °C overnight. The bacteria were diluted to an optical density (OD) of 0.05 at 600 nm. The samples were added to a 48 well plate, and 400 µL of the bacterial solution was added. The well plates were incubated in a static incubator for 24 hours. The Si substrates were removed from the well plate and washed three times in phosphate-buffered saline (PBS) to remove planktonic or loosely attached bacteria before being added to an Eppendorf tube containing 2 mL of PBS. To remove the biofilm from the substrates, the Eppendorf tube containing the retrieved substrates was vortexed for 30 s and ultrasonicated for 1 min before being vortexed for 30 s again. The solution was serial diluted and plated on lysogeny Agar plates (pH 7.5). The plates were incubated overnight, and the colony-forming units/mL (CFUs/mL) were calculated and plotted the next day.
